# Supplementary material for: Unique Brewing-Relevant Properties of a Strain of Saccharomyces jurei Isolated From Ash (Fraxinus excelsior)
Source: Front Microbiol. 2021 Mar 31;12:645271. doi: 10.3389/fmicb.2021.645271 (PMC8044551; doi:10.3389/fmicb.2021.645271)
Supplement: Supplementary file 1 [file Table_1.DOCX]

Supplementary Material

# Supplementary Data

*Saccharomyces jurei* TUM 629, partial ITS1-5.8S-ITS2 rDNA:

GAAGGATCATTAAAGAAATTTAATAATTTTGAAAATGGATTTTTTTGTTTTGGCAAGAGCATGAGCTTTTACTGGGCAAGAATACAAGAGATGGAGAGTCCAGTGGGGCCTGCGCTTAAGTGCGCGGTCTTACTAGGCTTGTAAGTTTCTTTCTTGCTATTCCAAACAGTGAGAGATCTTTGTGTTTTTGTTATAGGACAATTAAAACCGTTTCAATACAACACACTGTGGAGTTTTTATATCTTTGCAACTTTTTCTTTGGGCTTTCGAGCAATCGAGGCCCAGAGGTAACAAACACAAACAATTTTATTTATTCATTAAATTTTTGTCAAAAACAAGAATTTTCGTAACTGGAAATTTTAAAAATATTAAAAACTTTCAACAACGGATCTCTTGGTTCTCGCATCGATGAAGAACGCAGCGAAATGCGATACGTAATGTGAATTGCAGAATTCCGTGAATCATCGAATCTTTGAACGCACATTGCGCCCCTTGGTATTCCAGGGGGCATGCCTGTTTGAGCGTCATTTCCTTCTCAAACATTCTGTTTGGTAGTGAGTGATACTCTTTGGAGTTAACTTGAAATTGCTGGCCTTTTCATTGGATGTTTTTTTCCAAAGAGAGGTTTCTCTGCGTGCTTGAGGTATAATGCAAGTACGGTCGTTTTAGGTTTTACCAACTGCGGCTAATCTTTTTTGTACTGAGCGTATTGGAACGTTATCGATAAGAAGAGAGCGTCTAGGCGAACAATGTTCTTAAAGTTGACCTCAA

*Saccharomyces jurei* TUM 629, partial D1/D2 26S rDNA:

CAATAAGCGGAGGAAAAGAAACCAACCGGGATTGCCTTAGTAACGGCGAGTGAAGCGGCAAAAGCTCAAATTTGAAATCTGGTACCTTTGGTGCCCGAGTTGTAATTTGGAGAGGGCAACTTTGGGGCCGTTCCTTGTCTATGTTCCTTGGAACAGGACGTCATAGAGGGTGAGAATCCCGTGTGGCGAGGAGTGCGGTTCTATGTAAAGTGCCTTCGAAGAGTCGAGTTGTTTGGGAATGCAGCTCTAAGTGGGTGGTAAATTCCATCTAAAGCTAAATATTGGCGAGAGACCGATAGCGAACAAGTACAGTGATGGAAAGATGAAAAGAACTTTGAAAAGAGAGTGAAAAAGTACGTGAAATTGTTGAAAGGGAAGGGCATTTGATCAGACATGGTGTTTTGTGCCCTCTGCTCCTTGTGGGTAGGGGAATCTCGCATTTCACTGGGCCAGCATCAGTTTTGGTGGCAGGATAAATCCGTAGGAATGTAACTTGCTTCGGGAAGTATTATAGCCTGCGGGAATACTGCCAGCTGGGACTGAGGACTGCGACGTAAGTCAAGGATGCTTGCTTA

# Supplementary Tables

Supplementary Table S1: Genes in S. jurei NCYC 3947^T^ (accession number GCA_900290405) encoding potential maltotriose-transporting permeases

| **Gene** | **Chromosome** | **Start** | **End** | **Identity to S. cerevisiae S288C (%)** |
| --- | --- | --- | --- | --- |
| *MAL31*-like | LT986468.1 | 16347 | 18189 | 84,1 % |
| *MAL31*-like | LT986469.1 | 706843 | 704987 | 82,2 % |
| *MAL31*-like | LT986469.1 | 719396 | 717547 | 84,4 % |
| *MAL11*-like | LT986469.1 | 695682 | 693841 | 82,6 % |
| *IMA5*-like | LT986469.1 | 32730 | 31005 | 78,9 % |
| *IMA5*-like | LT986472.1 | 16533 | 14788 | 84,0 % |
| *IMA5*-like | LT986472.1 | 724412 | 726157 | 84,0 % |

# Supplementary Figures

#
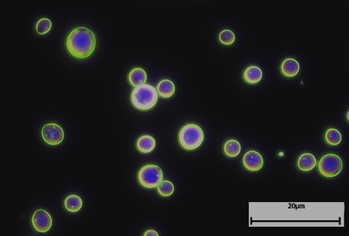


# Supplementary Figure 1. Cell morphology of *S. jurei* TUM 629 (48 h liquid culture in 12.4 °P Brewer’s wort from pale barley malt); dark field microscopy microscope (Axilob 5, Zeiss GmbH, Germany).


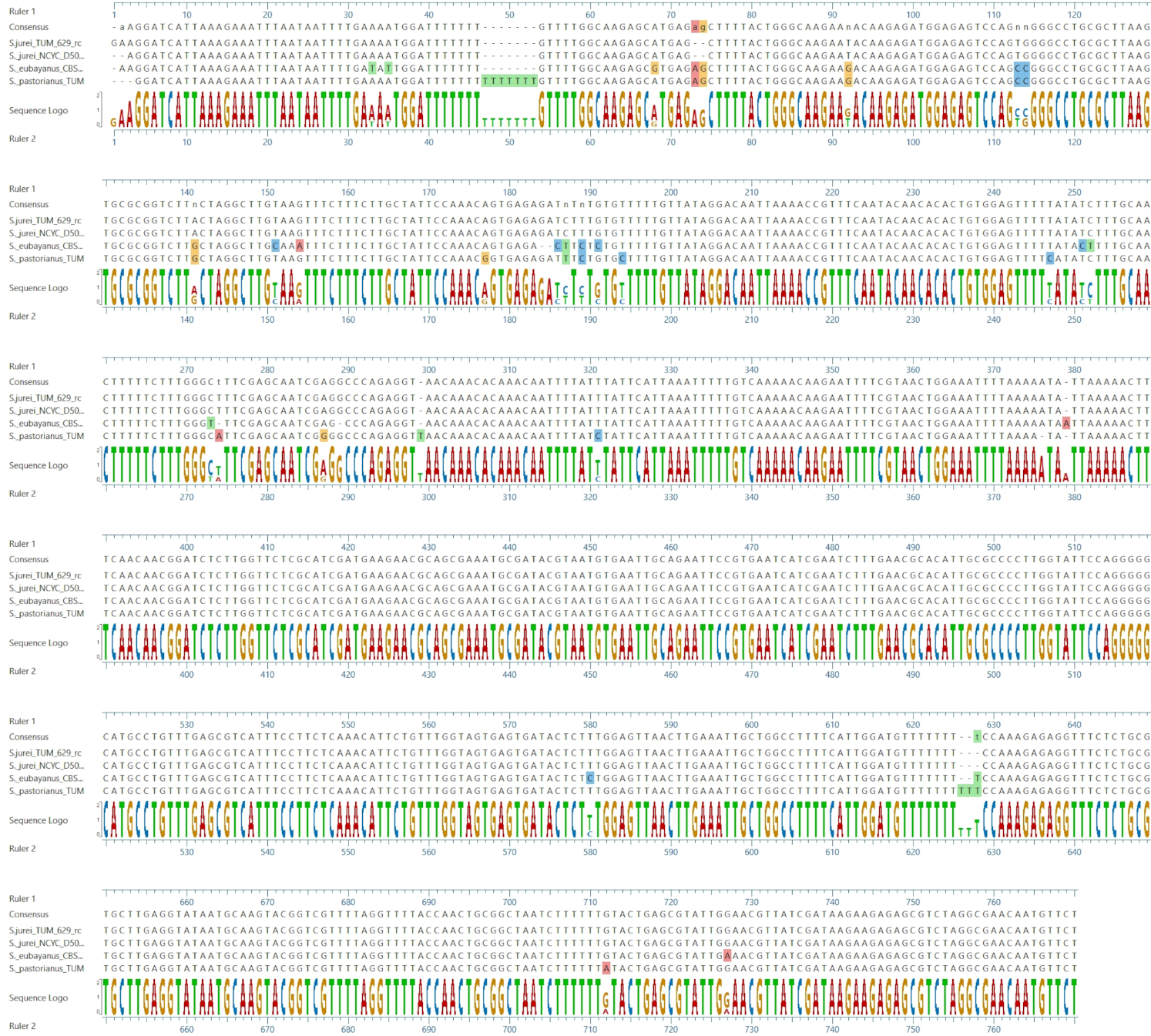


**Supplementary Figure 2.** Clustal W Alignment (multi sequence alignment) of *S. jurei* type strain NCYC 3947^T^, TUM 629, *S. eubayanus* C902 (CBS 12357^T^) and *S. pastorianus* TUM 34/70.


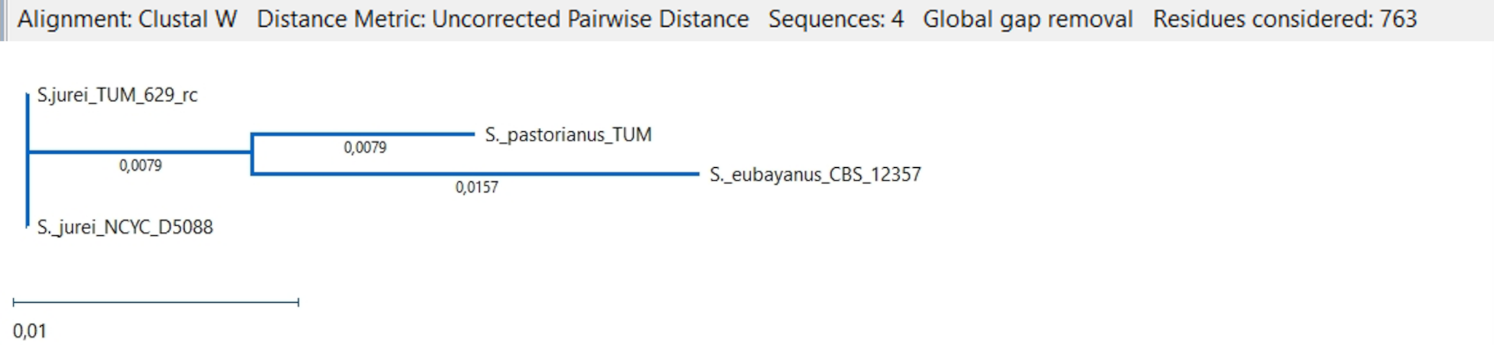


**Supplementary Figure 3.** Phylogenetic tree resulting from the Clustal W Alignment of *S. jurei* type strain NCYC 3947^T^, TUM 629, *S. eubayanus* C902 (CBS 12357^T^) and *S. pastorianus* TUM 34/70 showing the species identity of the newly isolated *S. jurei* strain.


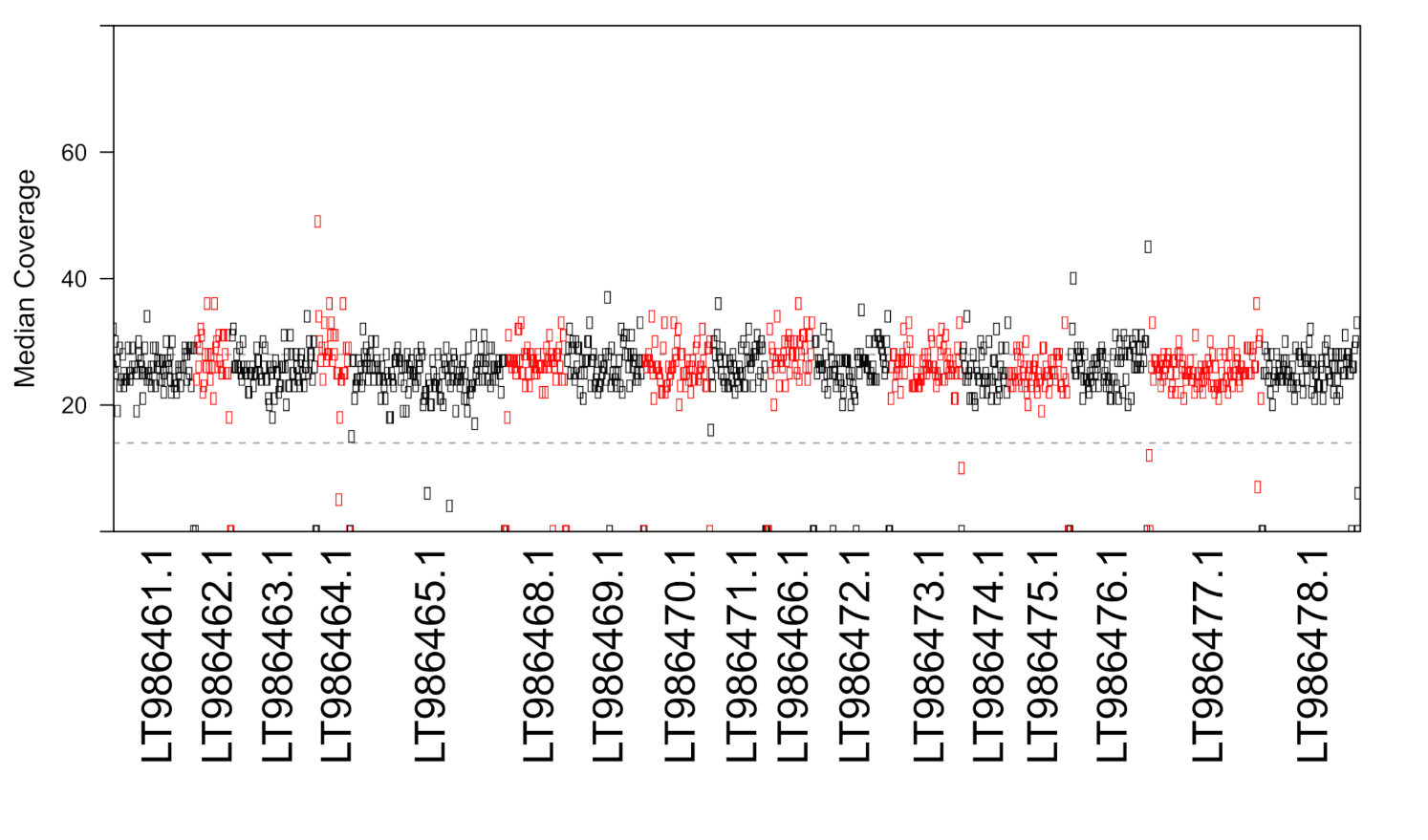


**Supplementary Figure 4.** The sequencing coverage (median coverage in 10 kbp windows) of reads from *S. jurei* TUM 629 aligned to a *S. jurei* NCYC 3947^T^ (NCBI accession number GCA_900290405; (Naseeb et al., 2018)) reference genome.


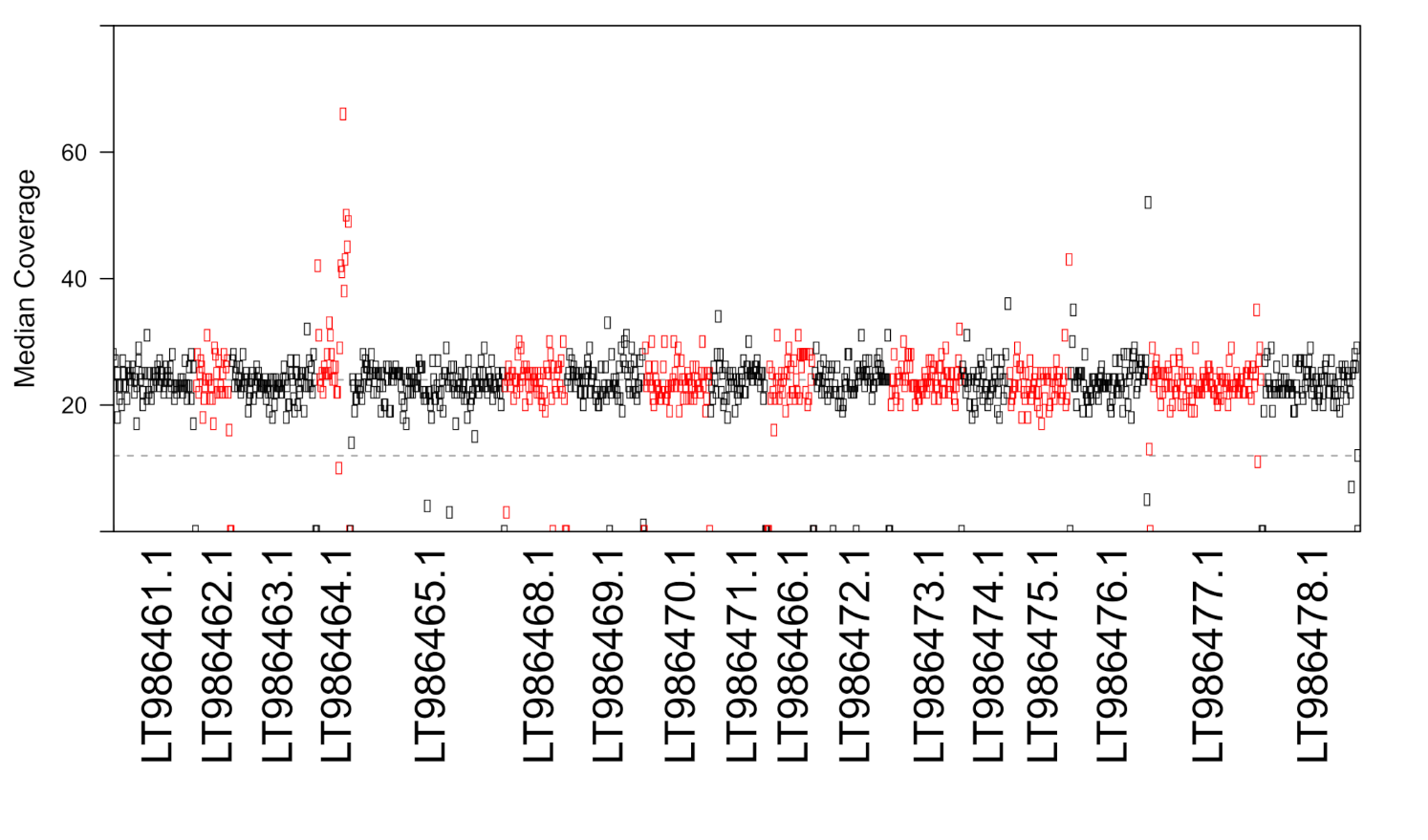


Supplementary Figure 5. The sequencing coverage (median coverage in 10 kbp windows) of reads from *S. jurei* VTT C-171003^T^ aligned to a *S. jurei* NCYC 3947^T^ (NCBI accession number GCA_900290405; (Naseeb et al., 2018)) reference genome.

# References

Naseeb, S., Alsammar, H., Burgis, T., Donaldson, I., Knyazev, N., Knight, C., et al. (2018). Whole Genome Sequencing, de Novo Assembly and Phenotypic Profiling for the New Budding Yeast Species *Saccharomyces jurei*. *G3 (Bethesda, Md.)* 8(9)**,** 2967-2977. doi: 10.1534/g3.118.200476.
